# Profile of patients with chronic obstructive pulmonary disease classified as physically active and inactive according to different thresholds of physical activity in daily life

**DOI:** 10.1590/bjpt-rbf.2014.0185

**Published:** 2016-09-22

**Authors:** Karina C. Furlanetto, Isabela F. S. Pinto, Thais Sant’Anna, Nidia A. Hernandes, Fabio Pitta

**Affiliations:** 1 Laboratório de Pesquisa em Fisioterapia Pulmonar (LFIP), Departamento de Fisioterapia, Universidade Estadual de Londrina (UEL), Londrina, PR, Brazil

**Keywords:** motor activity, pulmonary disease, chronic obstructive, energy metabolism, exercise, movement

## Abstract

**Objective:**

To compare the profiles of patients with chronic obstructive pulmonary disease (COPD) considered physically active or inactive according to different classifications of the level of physical activity in daily life (PADL).

**Method:**

Pulmonary function, dyspnea, functional status, body composition, exercise capacity, respiratory and peripheral muscle strength, and presence of comorbidities were assessed in 104 patients with COPD. The level of PADL was quantified with a SenseWear Armband activity monitor. Three classifications were used to classify the patients as physically active or inactive: 30 minutes of activity/day with intensity >3.2 METs, if age ≥65 years, and >4 METs, if age <65 years; 30 minutes of activity/day with intensity >3.0 METs, regardless of patient age; and 80 minutes of activity/day with intensity >3.0 METs, regardless of patient age.

**Results:**

In all classifications, when compared with the inactive group, the physically active group had better values of anthropometric variables (higher fat-free mass, lower body weight, body mass index and fat percentage), exercise capacity (6-minute walking distance), lung function (forced vital capacity) and functional status (personal care domain of the London Chest Activity of Daily Living). Furthermore, patients classified as physically active in two classifications also had better peripheral and expiratory muscle strength, airflow obstruction, functional status, and quality of life, as well as lower prevalence of heart disease and mortality risk.

**Conclusion:**

In all classification methods, physically active patients with COPD have better exercise capacity, lung function, body composition, and functional status compared to physically inactive patients.

## BULLET POINTS

- There are different cut-offs for identifying (i.e. classifying) physical (in)activity.
- The profile of COPD patients according to different cut-offs has never been investigated.
- Profile of active patients is similar regardless of the classification used.
- Active patients have clear health benefits compared to inactive patients.
- Interventions to modify sedentary behavior in COPD should be encouraged.

## Introduction

Chronic obstructive pulmonary disease (COPD) is characterized by chronic airflow limitation that is not fully reversible and consequent dyspnea^1^. In addition, patients with COPD present systemic manifestations of the disease, such as skeletal muscle dysfunction that leads to restrictions in exercise capacity, resulting in reduced levels of physical activity in daily life (PADL)^2^. Scientific evidence has clearly shown that patients with COPD are less active than healthy elderly patients^2^ ^,^ ^3^. Additionally, PADL is known to be closely related to a higher incidence of exacerbations and a higher mortality rate in this population^4^ ^-^ ^6^. Therefore, the detailed and accurate assessment of the PADL in these patients has generated scientific interest.

In order to conduct an objective and accurate evaluation of the level of PADL, the use of motion sensors is recommended. Motion sensors are portable devices that quantify the amount (and sometimes intensity) of physical activity performed by an individual in a given period of time^7^. This evaluation determines whether patients are considered physically active or inactive according to the minimum recommendations of physical activity practice^8^ ^,^ ^9^.

The physical activity recommendations published by the American College of Sports Medicine (ACSM) and the American Heart Association (AHA)^8^ suggest that physically active individuals are those who reach a minimum of 30 minutes per day of physical activity performed at least at moderate intensity, i.e., ≥3 Metabolic Equivalent of Task (METs) for all ages. However, in 2011, there was an update of the recommendations for physical activity by the ACSM^9^, and the moderate intensity during physical activity was determined according to age (>4 METs for individuals between 40 and 64 years of age and >3.2 METs for individuals ≥65 years of age). More recently, a study by van Remoortel et al.^10^ considered only bouts of physical activity of at least 10 minutes, without intervals, to detect if a patient with COPD reaches the recommendation of 30 minutes of physical activity of at least moderate intensity. These results suggested that physically active individuals are those who perform at least 80 minutes of physical activity with intensity ≥3 METs, without necessarily including the 10-minute bouts, characterizing them as continuous physical activity^8^ ^,^ ^10^. Therefore, there are different options to classify a patient as physically active or inactive, and the comparison between these different classifications has been little explored to date.

Taking into consideration the clinical relevance of correctly quantifying the level of physical activity of patients with COPD and knowing the different ways to classify the patient as physically active or inactive, this study aimed to compare the profile of patients with COPD who are considered physically active or inactive according to different classifications available in the literature.

## Method

### Study design and sample

In this cross-sectional study, 127 patients diagnosed with COPD according to the Global Initiative for Obstructive Lung Disease (GOLD)^1^ were initially included. In addition to the diagnosis of COPD, the following inclusion criteria of the study were considered: absence of bone, muscle, or nerve dysfunctions that could constrain PADL and other assessments; absence of severe or unstable heart disease; clinical stability (without occurrence of infections or exacerbations in the last three months); and not having practiced any kind of regular exercise program over the past year. The study was approved by the Research Ethics Committee of Universidade Estadual de Londrina (UEL), Londrina, PR, Brazil (061/06), and all subjects provided formal written consent. Patients who did not perform full PADL assessment (see below) were excluded.

### Physical activity assessment and classifications

PADL was objectively evaluated by a physical activity monitor (SenseWear Armband [SWA], BodyMedia Inc, Pittsburgh, PA, USA), which was validated for patients with COPD^11^ ^,^ ^12^. Patients were evaluated during two routine days of the week, 12 hours/day starting from the time of waking up, and the average of the two days was used for statistical analysis. The SWA is a biaxial accelerometer combined with physiological sensors, which offers as main outcomes the energy expenditure estimation and the time spent in physical activities stratified by intensity (‘active time’). The limits to determine if physical activity was performed in at least moderate intensity were determined according to the chosen recommendation as described below.

Three physical activity cut-offs based on previous literature were used in the same group of patients to classify patients as physically inactive or active: (1) Classification of 30 minutes based on age: the minimum recommendation is to achieve 30 minutes/day of physical activity at an intensity of at least 3.2 METs in patients ≥65 years of age and 4 METs in patients <65 according to the ACSM^9^; (2) Classification of 30 minutes regardless of age: the minimum recommendation is to achieve 30 minutes/day of physical activity at an intensity of at least 3 METs for all subjects according to the ACSM and AHA^8^ ^,^ ^9^; and (3) Classification of 80 minutes regardless of age: the minimum recommendation is to achieve 80 minutes/day of physical activity with an intensity of at least 3 METs. In the latter, the 10-minute blocks of physical activity without intervals (>3 METs) were based on the study by van Remoortel et al.^10^. The groups of physically active subjects included patients who reached the minimum recommendation of physical activity for each respective classification. Patients who did not reach the recommendations for each respective classification were classified as physically inactive.

### Secondary outcomes

Pulmonary function assessment was performed using the Spiropalm spirometer (Cosmed, Rome, Italy). The technique was carried out according to the guidelines of the American Thoracic Society^13^ and the reference values proposed for the Brazilian population by Pereira et al.^14^. The assessment of respiratory muscle strength was performed by measuring the maximum inspiratory and expiratory pressures (MIP and MEP, respectively) using a manometer, according to the technique described by Black and Hyatt^15^ and following the reference values for the Brazilian population described by Neder et al.^16^.

Functional exercise capacity was assessed using the six-minute walk test (6MWT), carried out in accordance with the standards recommended internationally in a 30-meter corridor^17^. The normal values used were those proposed for the Brazilian population by Britto et al.^18^.

Body composition was assessed by bioelectrical impedance (BIA 310, Biodynamics Corp, Seattle, WA, USA). This device uses patient data (gender, age, height and weight) to provide the following variables: fat mass (percentage of fat mass and fat mass in kg); body mass index (BMI); percentage of fat-free mass (FFM); fat-free mass index (FFMI); percentage of the ideal fat mass; body water; and target weight loss/gain. The assessment was performed according to the technique described by Lukaski et al.^19^, using the reference values described by Kyle et al.^20^ for fat mass and fat-free mass.

Peripheral muscle strength was assessed with the one repetition maximum test (1RM) of the knee extensors (i.e., quadriceps femoris), elbow flexors, and extensors. This test involves a trial and error procedure in which progressively heavier loads are raised until they exceed the capacity of the individual, thus determining the heaviest load that the individual is able to lift for one complete movement^21^.

Quality of life was assessed using a specific questionnaire for patients with COPD validated for Portuguese, the Saint George Respiratory Questionnaire (SGRQ)^22^. This questionnaire has three domains that evaluate the symptoms and activity and psychosocial impacts of the disease. Each domain has a maximum possible score; the scores of each answer are added up and the total is referred as a percentage of this maximum. A total score is also calculated based on the results of the three domains.

Functional status (limitations and ability to perform daily activities) was evaluated with two different tools: by the application of the London Chest Activity of Daily Living (LCADL)^23^ scale and the modified version of the Pulmonary Functional Status and Dyspnea Questionnaire (PFSDQ-M, Questionnaire on Dyspnea and Pulmonary Functional Status)^24^, both validated for the Portuguese language^23^. The LCADL is divided into four domains: personal care, household care, physical activity, and leisure. The score for each domain is calculated and the total score is the sum of all domains^23^. The PFSDQ-M questionnaire consists of three components: activity, dyspnea, and fatigue. In both instruments, higher values indicate worse functional status^24^.

The Medical Research Council (MRC) scale, also validated for Portuguese^24^, was used to evaluate the limitation by dyspnea in daily life. The scale consists of only five items, in which the patient chooses the item that corresponds to his/her limitation by breathlessness.

Comorbidities were assessed by a self-report questionnaire in which the patient indicates the presence or absence of medical diagnosis for a number of diseases (e.g. arthritis/osteoarthritis, hypertension, diabetes, osteoporosis, thyroid disease, vascular disease, and allergies).

The index known as BODE (BMI+Obstruction+Dyspnea+Exercise) was calculated using four variables: BMI (kg.m^–2^); degree of airway obstruction assessed with the forced expiratory volume in the first second (FEV_1_) in % of predicted values; dyspnea by the MRC scale (modified with the range from 0 to 4); and exercise capacity quantified by 6MWT distance in meters^25^. The total score ranges from zero to ten points, and higher values indicate higher risk of mortality.

### Statistical analysis

The Shapiro-Wilk test was used to analyze data normality. In case of normal distribution, results were reported as mean ± standard deviation; otherwise, data were expressed as median [interquartile range 25-75%]. To compare numerical variables of the two groups of patients (active and inactive, according to each cut-off), the Student t test or the unpaired Mann-Whitney test were used and statistical significance was determined as *P*<0.05. Categorical data were described as frequency and percentages and compared using the Chi-square test. Finally, one-way ANOVA was used to compare the characteristics of the three groups of patients classified as physically active according to those criteria, and the same was performed with the three groups classified as physically inactive. Statistical analysis was performed with Graph Pad Prism version 6.0.

## Results

### Sample

This convenience sample was initially composed of 127 patients; 23 of them were excluded for lack of full PADL data (patient did not use physical activity monitor properly or there were technical problems in the assessment). The final sample (n=104) had mean age of 66±8 years, FEV_1_ 42±16%predicted, and BMI 26±6 kg.m^–2^. There was no statistical difference when the sample (n=104) was compared with the 23 excluded patients (*P*>0.05 for all variables). Of the analyzed patients, 63% were men, 72% were literate, 80% were ex-smokers, and 3% used long-term oxygen therapy. The demographic and anthropometric characteristics of the patients included in the study are described in Table 1. Eighty-one percent reported at least one comorbidity, while the median [IQR25-75%] of comorbidities per patient was 1[2-3]. Additionally, 31% presented arthritis, 19% heart disease, 54% hypertension, 18% diabetes, 9% osteoporosis, 10% thyroid-related diseases, 38% vascular disorders, and 38% allergies.

**Table 1:** Characteristics of patients with COPD enrolled in the study.

**Variable**	**(n = 104)**
**Gender, M/F (%)**	66/38 (63/37)
**Age (years)**	66±8
**Weight (kg)**	67±16
**Height (m)**	1.60±0.09
**BMI (kg.m^–2^)**	26±6
**FEV_1_ (L)**	0.98 [0.70-1.34]
**FEV_1_ (% pred)**	42±16
**6MWT (m)**	544±42
**6MWT (%)**	82 [72-94]
**MIP (cmH_2_O)**	70 [50-80]
**MIP (% pred)**	69±26
**MEP (cmH_2_O)**	100 [77-120]
**MEP (% pred)**	96 [81-122]
**MF-QF (kg)**	15±6
**MF-TB (kg)**	10±4
**MF-BB (kg)**	12±4
**GOLD, I/II/III/IV (%)**	1/30/42/30 (1/29/41/29)
**BODE, I/II/III/IV (%)**	27/34/33/10 (26/33/32/9)
**mMRC (pts)**	3 [2-3]
**LCADL total (pts)**	21 [16-28]
**SGRQ total (pts)**	52 [40-64]
**PFSDQ-M** a **(pts)**	18 [8-31]
**PFSDQ-M** b **(pts)**	16 [7-30]
**PFSDQ-M** c **(pts)**	22 [10-37]

Values are described as mean± standard deviation or median [interquartile range 25-75%] or absolute frequency (relative). M: male; F: female; BMI: body mass index; FEV_1_: forced expiratory volume in the first second; 6MWT: six-minute walking test; MIP: maximal inspiratory pressure; MEP: maximal expiratory pressure; MF: muscle force; QF: quadriceps femoris; TB: triceps brachii; BB: biceps brachii; GOLD: Global Initiative for Chronic Obstructive Lung Disease; BODE (BMI; Obstruction; Dyspnea; Exercise): patients classified in quartile of grouped BODE I, II, III, and IV^25^. mMRC: modified version of Medical Research Council; LCADL: London Chest Activity of Daily Living scale; SGRQ: Saint George Respiratory Questionnaire; PFSDQ-M: modified version of the Pulmonary Functional Status and Dyspnea questionnaire.

a Dyspnea Domain PFSDQ-M;

b Fatigue Domain PFSDQ-M;

c Activity Domain PFSDQ-M.

### Profile of physically active vs inactive patients

In the 30-minute classification based on age, the proportion of active versus inactive patients was 35% vs 65%, while this proportion was 56% vs 44% in the 30-minute classification regardless of age and 27% vs 73% in the 80-minute classification regardless of age. There was statistical difference between the proportion of individuals considered active and inactive in all three forms of classification (*P*<0.0001).

When compared to the inactive group in three different classifications, the active group showed lower weight, BMI, percentage of fat mass and score in the personal care domain of the LCADL scale, besides presenting a higher percentage of fat-free mass, forced vital capacity and distance walked in the 6MWT (Table 2). Additionally, if we take into consideration a statistical difference in two classifications, physically active patients also had higher values of 1RM of knee extensors, maximum expiratory pressure and forced expiratory volume in the first second. Furthermore, physically active patients had lower (i.e. better) functional status total score and quality of life in the activity domain of the SGRQ, lower incidence of stable heart disease and lower (i.e., better) score in the BODE (BMI+Obstruction+Dyspnea+Exercise) index (Table 3). Table 3 also shows other characteristics of patients with significant differences in only one of those classifications. There were no differences among the groups of patients classified as physically active according to the three criteria (*P*>0.05 for all) or among the groups of patients classified as physically inactive.

**Table 2:** Differences among patients with COPD classified as physically active or inactive according to all 3 cut-off points of physical activity/inactivity.

**Variable**	**30 min based on age**		**30 min regardless of age** a		**80 min regardless of age** a	
	**Active** **(n = 36)**	**Inactive** **(n = 68)**	**Active** **(n = 58)**	**Inactive** **(n = 44)**	**Active** **(n = 28)**	**Inactive** **(n = 74)**
**Weight (kg)**	60±12	71±16^*^	63±15	72±16^*^	57[48-64]	71[58-80]^*^
**BMI (kg.m^–2^)**	23±4	28±6^*^	24±15	28±6^*^	23±4	27±6^*^
**FFM (%)**	68[62-76]	60[55-69]^*^	65[58-75]	60[54-68]^*^	71[62-78]	61[56-69]^*^
**FM (%)**	28[23-36]	35[29-39]^*^	29[24-35]	35[29-41]^*^	28[22-32]	35[28-390]^*^
**6MWT (m)**	567[536-590]	535[509-557]^*^	555 ± 38	530 ± 43^*^	569 ± 36	535 ± 41^*^
**6MWT (%)**	88[81-95]	79[70-91]^*^	87[79-98]	75[68-83]^*^	87[80-97]	82[71-93]^*^
**FVC (L)**	2.6±0.8	2.0±0.6^*^	2.5±0.8	1.9±0.6^*^	2.7±0.9	2.0±0.6^*^
**FVC (%)**	60[0-71]	59[48-71]*	69[55-84]	59[46-68]^*^	76[62-86]	60[48-72]^*^
**LCADL - Personal care (pts)**	5[4-6]	6[5-9]^*^	5[4-7]	7[5-9]*	5[4-6]	6[5-8]^*^

Values are described as mean ± standard deviation or median [interquartile range 25-75%]. 30 min based on age: 30 min/day of physical activity with intensity 3.2 METs if ≥65 years old and 4 METs if <65 years old; 30 min regardless of age: 30 min/day of physical activity with intensity >3.0 METs regardless of age; 80 min regardless of age: 80 min/day of physical activity with intensity >3.0 METs regardless of age. BMI: body mass index; FFM: fat-free mass; FM: fat mass; 6MWT: six-minute walking test; FVC: forced vital capacity.

* P≤0.05 vs active;

a Two patients had missing data for physical activity in daily life with an intensity of at least 3 METs.

**Table 3:** Differences among patients with COPD classified as physically active or inactive which reached a statistically significant difference in only one or two cut-off points of physical activity/inactivity.

**Variable**	**30 min based on age**		**30 min regardless of age** a		**80 min regardless of age** a	
	**Active** **(n = 36)**	**Inactive** **(n = 68)**	**Active** **(n = 58)**	**Inactive** **(n = 44)**	**Active** **(n = 28)**	**Inactive** **(n = 74)**
**Age (years)**	65±9	66±8	64±8	68±8^*^	63±9	66±8
**FFMI (kg/m^2^)**	16±2	17±2^*^	16±2	17±2	16±1	16±2
**FEV_1_(L)**	1.2[0.8-1.6]	0.9[0.6-1.2]	1.2[0.8-1.5]	0.8[0.6-1.0]^*^	1.8[1.2-2.8]	0.9[0.7-1.3]
**FEV_1_ (% pred)**	45[35-55]	39[26-50]^*^	43[33-55]	34[25-49]^*^	45[30-55]	40[26-53]
**MF-QF (kg)**	17±6	14±6*	16±6	13±7^*^	17±6	14±6
**MF-TB (kg)**	12[10-15]	11[8-14]	13[10-16]	10[7-13]^*^	13 ± 5	11 ± 4^*^
**MF-BB (kg)**	11±4	9±4	8[5-11]	8[6-12]^*^	10[8-14]	10[6-13]
**MEP (% pred)**	89[72-112]	105[85-132]^*^	93[77-118]	104[84-125]	87[67-103]	106[85-130]^*^
**mMRC (pts)**	3[1-3]	3[2-3]	2[1-3]	3[2-3]^*^	3[1-3]	3[2-3]
**LCADL (pts)**	18[15-26]	23[16-29]^*^	20[17-28]	17[16-29]	18[15-26]	23[16-29]^*^
**SGRQ-Activity (pts)**	60[48-74]	70[53-81]^*^	61±19	66±22	60[48-73]	68[49-81]^*^
**PFSDQ-M Dyspnea (pts)**	16[6-27]	20[10-33]	16[8-28]	19[10-34]	11[6-20]	20[12-33]^*^
**PFSDQ-M Fatigue (pts)**	12[6-25]	18[8-32]	14[8-28]	18[7-32]	9[5-17]	19[8-31]^*^
**BODE, I+II/III+IV (%)**	27/9 (75/25)	34/34(50/50)^*^	43/15(74/26)	17/27(39/61)^*^	21/7(75/25)	39/35(53/47)
**Heart disease, Y (%)**	3(8)	15(25)	7(10)	12(28)^*^	1(3)	16(24)^*^

Values are described as mean ± standard deviation or median [interquartile range 25-75%]. Categorical variables expressed in absolute frequency (relative): BODE I+II/III+IV: patients classified in quartile of grouped BODE I and II (≤4 points) compared with BODE III and IV (≥ 5 points); Heart Disease, Yes: Presence of self-referred stable heart disease. 30 min based on age: 30 min/day of physical activity with intensity 3.2 METs if ≥65 years old and 4 METs if <65 years old; 30 min regardless of age: 30 min/day of physical activity with intensity >3.0 METs regardless of age; 80 min regardless of age: 80 min/day of physical activity with intensity >3.0 METs regardless of age. FFMI: fat-free mass index; FEV_1_: Forced expiratory volume in the first second; MF: muscle force; QF: quadriceps femoris; TB: triceps brachii; BB: biceps brachii; MEP: maximal expiratory pressure; mMRC: modified version of Medical Research Council; LCADL: London Chest Activity of Daily Living scale; SGRQ: Saint George Respiratory Questionnaire; PFSDQ-M: modified version of the Pulmonary Functional Status and Dyspnea questionnaire.

* *P*≤0.05 vs active.

a Two patients had missing data of physical activity in daily life with an intensity of at least 3 METs.

## Discussion

This study showed that patients with COPD classified as physically active, regardless of the classification (i.e., cut-off), have lower weight, BMI, percentage of fat mass, and score in the personal care domain of the LCADL scale as well as have a higher percentage of fat-free mass, forced vital capacity, and 6MWT distance compared to physically inactive patients. In addition, physically active patients have higher values for peripheral and expiratory muscle strength, better (i.e. lower) scores for functional status and quality of life in the activity domain of the SGRQ, and lower airway obstruction, prevalence of stable heart disease, and mortality risk.

The comparison between active and inactive patients was previously investigated by Pitta et al.^7^. That study showed that COPD patients classified as physically inactive had worse exercise capacity and pulmonary function and higher scores in the BODE index. The same differences were found in the present study, even when using different ways to distinguish physically inactive patients from active ones. In the study by Pitta et al.^7^, the walking time above 30 minutes was used as the cut-off point, regardless of the intensity of physical activity, whereas a novelty of the present study is that it was possible to characterize and identify the differences between physically active and inactive patients with COPD by using three different classifications described in the scientific literature that consider activities of at least moderate intensity.

The observed differences in body composition between physically active and inactive patients were demonstrated in a previous study of Monteiro et al.^26^. The relationship between obesity and physical activity in daily life in this population showed that physically inactive patients had higher body weight and lower fat-free mass. The results of the present study confirmed that, regardless of the classification method, physically inactive patients had worse body composition when compared to active patients.

In a previous study about the profile of Brazilian patients with COPD^3^, the movement intensity in daily life was positively correlated with the 6MWT (r=0.42) and negatively with the personal care domain and total score in the LCADL scale, the MRC, the BODE index, and age (–0.32 < r < –0.58 for all). Additionally, walking time was negatively correlated with the MRC scale (r=–0.31), BODE index (r=–0.30), and age (r=–0.43)^3^. In the present study, physically inactive patients also had worse scores in the personal care domain of the LCADL scale and the BODE index, which respectively confirm the more limited functionality in activities of daily living and higher risk of mortality. On the other hand, limitation by dyspnea assessed by the MRC scale was higher in most physically inactive patients when using the 30-minute classification regardless of age but not the 30-minute classification based on age and the 80-minute classification regardless of age. These results support the concept that dyspnea assessed by a scale with a rather small range (i.e: from 0 to 4) is not highly associated with PADL outcomes in patients with COPD, as previously shown^3^ ^,^ ^27^.

Among the evaluated comorbidities, patients classified as physically inactive showed higher presence of self-reported heart disease compared to physically active patients in two classifications. This was not a surprising result since Watz et al.^28^ concluded that cardiac dysfunction is associated with physical inactivity in patients with COPD. Therefore, due to this combination, patients with heart disease associated with chronic lung disease should be priority targets in the search for interventions that aim to reverse the physical inactivity.

In addition to the differences observed between the three methods of classifying physical activity (Table 2), other differences were found in the comparison between physically active and inactive patients considering only one or two of the recommendations (Table 3). Factors such as the degree of obstruction (FEV_1_% predicted), peripheral muscle strength of knee and elbow extensors, expiratory muscle strength (MEP), functionality (LCADL), and quality of life (SGRQ activity domain) were better in physically active patients in two classifications, and may also be considered as indicators of profile differences between active and inactive patients.

A limitation of the study is the fact that there are other ways of classifying individuals as physically active or inactive in the scientific literature. Hartman et al.^29^ showed that the application of seven different physical activity recommendations in the same population led to large differences in the classification of patients with COPD as sufficiently physically active or not; however, that study aimed to compare the different classifications of physical activity and not the characteristics of physically active or inactive patients, as in the present study. The choice of the three classifications proposed in this study was due to the fact that these are commonly used in the recent literature aimed at objectively quantifying the level of PADL in COPD. Moreover, the proportion of patients classified as physically active or inactive in this study was not similar in the three classifications, and although other options are available in the literature^29^, the chosen classifications were sensitive to detect these differences. Another limitation is the self-report assessment of comorbidities, since this may under/overestimate the proportion of patients with stable heart disease and other diseases. However, the authors did not have access to other diagnostic methods for these comorbidities, and this self-reported evaluation is commonly used in studies in the scientific literature to investigate the profile of patients with COPD^3^ ^,^ ^26^. Finally, despite the fact that at least two days of physical activity assessment has shown acceptable reliability in patients with COPD^2^ ^,^ ^30^, it has been recommended that at least 5 days of physical activity assessment are necessary for patients with mild disease^31^. Although only two days of assessment can be seen as a limitation, the present sample was composed largely of severe patients, and therefore it appears unlikely that the results were influenced by this methodological characteristic. Future studies should include more patients with mild disease and at least five days of assessment of physical activity in daily life.

## Conclusion

Regardless of the classification used to identify and classify patients with COPD as physically active or inactive, the physically active patients have better exercise capacity, body composition, lung function, and functional status compared to the physically inactive patients. These results indicate the clear health benefits physical activity for patients with COPD and further motivate researchers to seek interventions to modify the sedentary behavior observed in a large number of patients with COPD.
